# Valproate-Induced Encephalopathy Presenting at Therapeutic Blood Concentrations: A Case Report and Literature Review

**DOI:** 10.7759/cureus.33559

**Published:** 2023-01-09

**Authors:** Gautham Pavar, Nicole Xu, Kinan Sawar, Vichar Trivedi, Diane L Levine

**Affiliations:** 1 Medicine, Wayne State University School of Medicine, Detroit, USA; 2 Ophthalmology, Wayne State University, Detroit Medical Center, Detroit, USA; 3 Internal Medicine, Wayne State University, Detroit Medical Center, Detroit, USA

**Keywords:** seizure prophylaxis, valproic acid toxicity, cyp2c9, l-carnitine, urea cycle deficiency, acute hyperammonemic encephalopathy, valproate

## Abstract

Patients presenting with hyperammonemic encephalopathy are likely to have hepatic encephalopathy. However, valproate (an anticonvulsant and mood stabilizer) can also cause hyperammonemic encephalopathy and belongs on the differential for patients taking it, especially if there are recent contributory medication changes. We present a case report of a 61-year-old woman with valproate-induced hyperammonemic encephalopathy but with an initial valproate level within the therapeutic range (50-100 mcg/dL). After withholding valproate and before additional treatment could be initiated, she became fully alert and oriented. We present a literature review exploring valproate toxicity and treatment. Our case shows that clinical suspicion for valproate-induced hyperammonemic encephalopathy is warranted even if the valproate level is within the therapeutic range.

## Introduction

Valproate is an anti-seizure medication also indicated for anxiety, mood disorders, and migraines. Unfortunately, while it treats many different pathologies, it also has many different toxicities. Severe toxicities include hepatic failure, hemorrhagic pancreatitis, encephalopathy, and neural tube defects in utero. Less fatal toxicities include tremors, dizziness, thrombocytopenia, depression, nystagmus, headache, and abdominal pain [1]. The challenge in using valproate is that blood serum concentration correlates poorly with both treatment efficacy and toxicity [1]. This case is rare in that it explores valproate-induced encephalopathy at therapeutic valproate serum levels. 

This article was previously presented as a poster at the 2022 Michigan American College of Physicians Annual Scientific Meeting on October 15, 2022.

## Case presentation

Our patient was a 61-year-old woman with cognitive developmental delay, schizophrenia, seizure disorder, and hypertension who presented to the emergency department for “slurring words and not acting like self.” All history was gathered from the family since the patient could not answer questions herself. Three weeks before admission, she was prescribed valproate for seizure control. Her home medications also included atenolol, haloperidol, cogentin, and trazodone. Over the previous 10 days, she had been clumsier than usual, grabbing onto railings to hold herself steady, and dropping medications and food from her hands. Additionally, she would fall forward or backwards while trying to sit still and would occasionally have episodes where her extremities became rigid. The family denied loss of consciousness, urinary incontinence, and seizure-like episodes over this time frame but affirmed decreased appetite.

The patient was somnolent during the evaluation. With stimulation, she would answer a simple “yes” to all questions. During the physical exam, she was afebrile, saturating well on room air, and mildly hypertensive. Her conjunctiva were anicteric. Heart and lung exams were normal. An abdominal exam showed no signs of liver disease. During the mental status exam, she showed no insight or judgment into her condition and health. Neurologically, the patient was oriented only to herself with a slight postural tremor present with extended arms. Cranial nerves 2-6 were intact; extraocular movements were assessed by directing the patient’s attention toward different areas of her visual field. Other nerves were unable to be assessed. Motor strength and reflexes were intact for all extremities. Sensation and ambulation could not be assessed. The skin exam was negative for spider angiomata.

**Figure 1:**
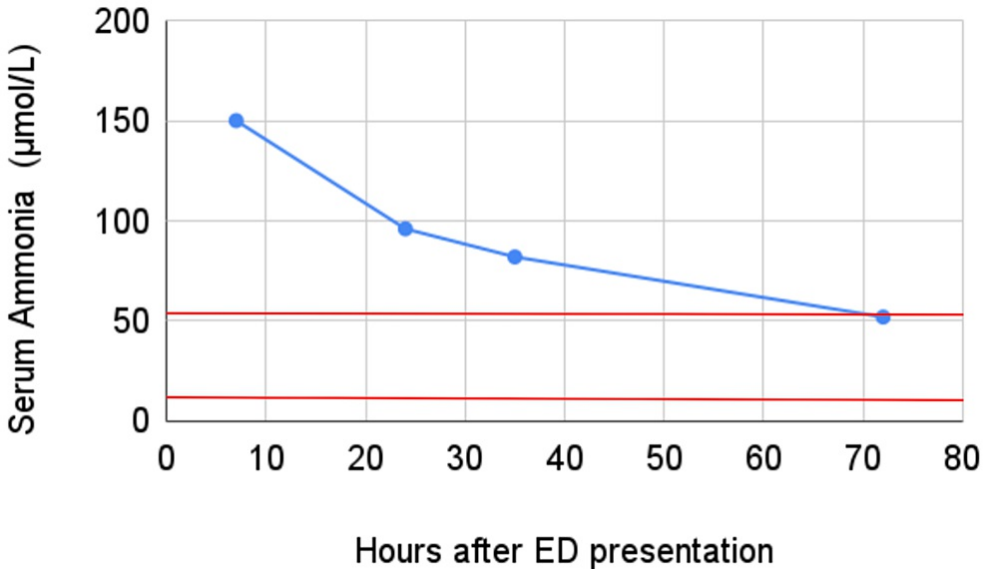
Serum ammonia levels measured during the patient’s hospital stay. Ammonia levels were no longer drawn after they reached the normal range (shown in red).

Electrolytes and all liver tests including transaminases, alkaline phosphate, and bilirubin were normal. Mild thrombocytopenia at 142,000 platelets per mcL was present. The patient was not anemic. Prothrombin time (PT) and partial thromboplastin time (PTT) were within normal limits. Ammonia was elevated at 150 μmol/L (normal range, 11-53 μmol/L) (Figure 1). Imaging studies (head computed tomography, magnetic resonance imaging, and chest X-ray) were all normal. Admission urinalysis was normal.

Given the clinical picture above, we suspected valproate-induced hyperammonemic encephalopathy and stopped all home medications. We empirically started the patient on lactulose to reduce ammonia levels. EEG confirmed our working diagnosis by showing generalized slowing indicative of cerebral dysfunction consistent with metabolic or hypoxic encephalopathy.

**Figure 2:**
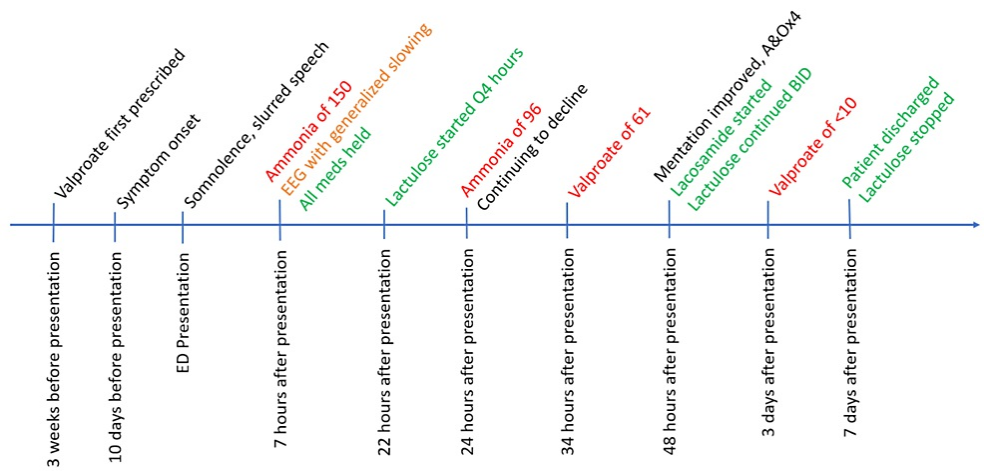
Timeline of events, important lab findings, and interventions from patient presentation to discharge.

The valproate level was 61 mcg/mL total and 10.7 mcg/mL free, ​​this was in the therapeutic range of 50-100 mcg/mL. The toxic range is >100 mcg/mL. Forty-eight hours later, the patient’s ammonia level trended down and the patient’s mentation improved; she was no longer somnolent, and she was alert and oriented to person, place, time, and event. The team replaced valproate with lacosamide in her home medications. A timeline of these events is shown in Figure 2.

## Discussion

Medication and valproate toxicity

Medication toxicity can be classified into two main classes: predictable and unpredictable. Predictable toxicities include medication side effects like NSAIDs causing gastrointestinal bleeding [2]. Unpredictable toxicities include medication allergies or genetic susceptibilities like CYP450 mutations altering the metabolism of drugs. Diagnosis is dependent on a thorough history gained from the patient and bystanders and also the physical exam [2]. Laboratory studies including toxicology studies, anion gaps, and osmolar gaps can also help narrow down the etiology. 

There are several ways to treat medication toxicity. To prevent further absorption, decontamination agents like activated charcoal can be used. Activated charcoal makes compounds like aspirin bind to itself instead of to the walls of the gastrointestinal tract [3]. Dialysis can be used in cases of severe toxicity to remove certain drugs from the bloodstream. For example, it can be used in methanol toxicity [3]. In some cases, clinicians have access to antidotes, which can prevent or reverse the toxicity of specific compounds; for example, fomepizole is an antidote for methanol poisoning [3].

Regarding valproate toxicity, the diagnosis can be challenging to make since the symptoms are nonspecific. In the context of central nervous system signs, the patient may experience lethargy progressing to drowsiness, coma, and even fatal cerebral edema [4]. Hyperammonemia may be seen but is not specific for this toxicity since many patients can have hyperammonemia and be otherwise healthy [4]. Additionally, the level of serum ammonia correlates poorly with the level of encephalopathy caused by valproate [4]. Patients with valproate toxicity can have metabolic acidosis, hypernatremia, and/or hypocalcemia. Hepatotoxicity can be characterized by an elevated alanine transaminase with low gamma-glutamyl transferase but is not always present in valproate toxicity as in the case of our patient.

**Figure 3:**
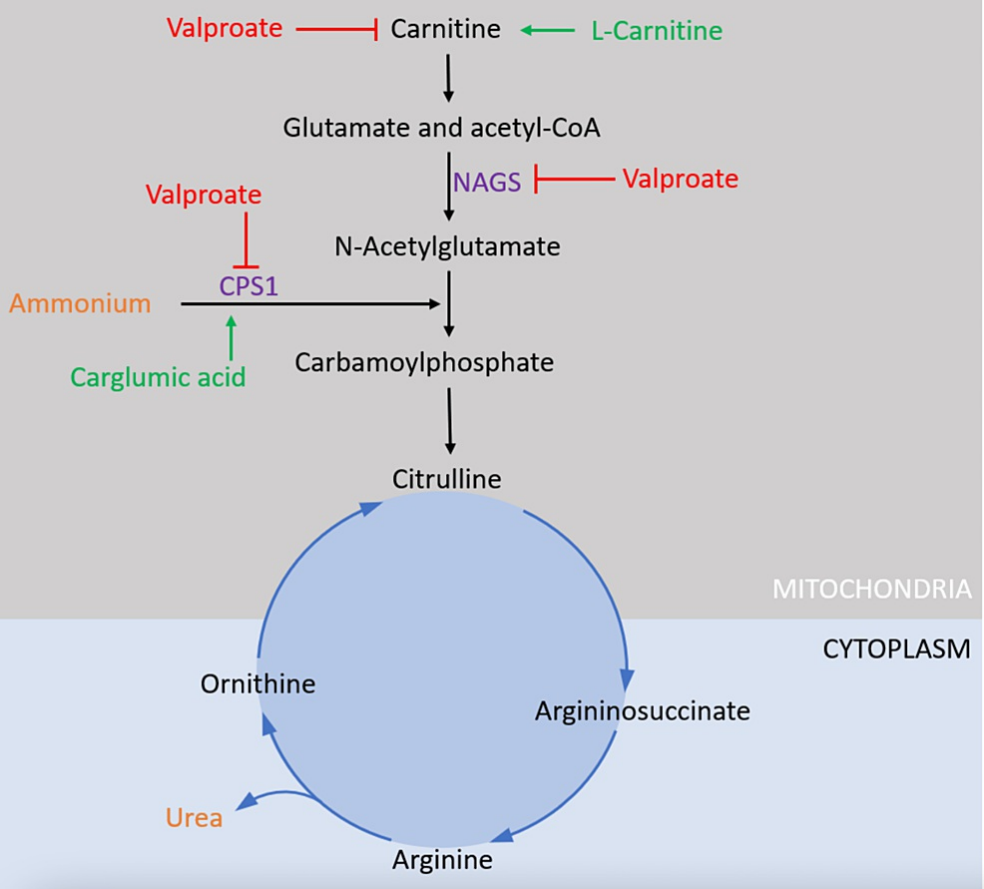
How valproate inhibits the urea cycle and how treatments counteract it. NAGS: N-acetylglutamate synthase; CPS1: carbamoyl phosphate synthetase. The figure originates from the article by Baumgartner et al. [7]. Reprinted with permission from the American Journal of Psychiatry. All rights reserved. Permission number: PL19242.

There are reports of valproate causing hyperammonemia at therapeutic levels [5]. One of the potential explanations for this is genetic susceptibility. Cytochrome P450 mutations can be culprits, especially given valproate’s reliance on CYP2C9 for clearance; however, other culprits include urea cycle disorders. They are inherited in an autosomal recessive fashion. Carriers usually have one functional gene which is sufficient enough for daily life [6]. However, valproate can inhibit the cycle at many stages (Figure 3). It can deplete carnitine, a necessary substrate, and inhibit carbamoyl phosphate synthase (CPS), both of which are necessary to make carbamoyl phosphate, which is the main input of the urea cycle [7]. For example, if a patient is already deficient in functional enzymes for CPS, even a subtherapeutic level of valproate can make them hyperammonemic. Kankananarachchi et al. (2021) published a case report showing exactly that; they describe a child with only one functional copy of CPS who started taking valproate for epilepsy and became encephalopathic as a result [8].

Current literature on treating valproate toxicity

The first-line treatment of valproate toxicity is L-carnitine. The rationale for its use is based on Figure 3. Since valproate depletes the body’s stores of L-carnitine, the theory goes that replenishing it will keep the urea cycle active and consequently keep ammonia levels low. This mechanism is theorized to reduce brain edema and treat valproate-induced encephalopathy [7,9]. There is data supporting using L-carnitine as chronic supplementation in patients prescribed valproate [10,11]. However, the literature is mixed on the efficacy of using L-carnitine for acute toxicity. Nguyen et al. note that while L-carnitine reduces peak serum lactate levels in acute valproate toxicity, it does not speed up the return of blood lactate to normal levels nor help in valproate clearance [12], meaning that L-carnitine may help reduce the extent of organ damage in severe valproate toxicity, but may not improve prognosis and length of hospital stay.

We could not identify literature comparing L-carnitine treatment against other therapies for valproate toxicity in patients with mildly elevated or normal valproate levels. These kinds of trials would be helpful since mild to moderate valproate toxicity treated with medication cessation and conservative treatment has a good prognosis already. Shadnia et al. published a case series of 316 patients who only received conservative measures like airway protection and IV fluids since IV L-carnitine was not available. Most of their patients recovered fully and only 14 had poor outcomes and required intubation and hemodialysis with extended hospital stay [13]. However, while L-carnitine does have a package insert warning of increased seizure risk, there are no reports in the literature of L-carnitine-induced seizures [14]. Controlled trials comparing L-carnitine to conservative management are unlikely to be performed. Given the excellent safety profile of L-carnitine, it would be unethical to deny patients L-carnitine when it may be effective and lacks evidence of side effects.

Since it is the safest medication, L-carnitine is the first-line treatment. Nevertheless, other compounds have been studied, such as meropenem. Unlike L-carnitine, meropenem has been shown to reduce serum valproate concentrations by preventing reabsorption in the gastrointestinal tract by inhibiting a specific reuptake enzyme (acylpeptide hydrolase), thereby reducing valproate’s half-life [15,16]. However, we were only able to find recent case reports showing use in the clinical environment: For example, Sanivarapu et al. published a case of valproate-induced encephalopathy refractory to IV carnitine but not to meropenem [16]. It is not the first-line treatment for valproate toxicity because of its side effects of diarrhea, rash, and decreased seizure threshold [17,18]. The last adverse effect would not be ideal in patients receiving valproate for seizure prophylaxis. In addition, widespread use of meropenem would hamper antibiotic stewardship. 

Carglumic acid is another less-studied medication for valproate toxicity. It is a structural analog of N-acetylglutamate (NAG) that activates carbamoyl phosphate synthetase 1 (CPS1) in the urea cycle (Figure 3). It has traditionally been used in pediatric patients for the treatment of hyperammonemic encephalopathy due to valproate toxicity [19]. However, the efficacy of carglumic acid for valproate toxicity is unclear, especially in adults, and there have not been any randomized controlled trials using carglumic acid for valproate toxicity [19].

Dialysis can be an option if medication is ineffective or contraindicated. Acute valproate toxicity is amenable to dialysis since valproate is mostly water soluble and has a low volume of distribution [20]. Dialysis can also be combined with L-carnitine for added effect [21]. However, dialysis is not useful in all cases of valproate toxicity. In cases like our patient’s, where serum valproate is within therapeutic levels, the drug is bound to proteins and unable to be dialyzed. Dialysis is effective if the starting valproate level is over 900 mg/dL [20]. Additionally, dialysis can predispose the patient to “rebound toxicity” where blood cleared out of toxins too quickly can have toxins diffusing back into it from other fluid compartments [20].

In addition to the definitive treatments mentioned above, naloxone can be used as a symptomatic treatment to prevent respiratory depression [20].

In summary, L-carnitine is the first-line treatment for acute valproate toxicity due to its safety; however, it does not help decrease serum valproate levels. In more severe poisonings where valproate clearance must be expedited, meropenems and hemodialysis are available as options.

## Conclusions

One severe toxicity of valproate is encephalopathy, which can even occur at therapeutic valproate serum levels and can be associated with hyperammonemia. Good history-taking and medication reconciliation are necessary for diagnosis. Genetic mutations in cytochrome P450 enzymes or urea cycle enzymes can make patients susceptible to encephalopathy at therapeutic valproate serum levels. Once valproate toxicity is suspected, it makes sense to start L-carnitine given its benign side effect profile. L-carnitine should be followed up with further treatment if encephalopathy is still present. Given the authors’ experience, L-carnitine as the first-line treatment for valproate toxicity is not as widely known as it should be, indicating the need for further dissemination.
